# Measuring macromolecular size distributions and interactions at high concentrations by sedimentation velocity

**DOI:** 10.1038/s41467-018-06902-x

**Published:** 2018-10-24

**Authors:** Sumit K. Chaturvedi, Jia Ma, Patrick H. Brown, Huaying Zhao, P. Schuck

**Affiliations:** 1 Dynamics of Macromolecular Assembly Section, Laboratory of Cellular Imaging and Macromolecular Biophysics, National Institute of Biomedical Imaging and Bioengineering, National Institutes of Health, Bethesda, MD 20892 USA; 2 Present Address: Department of Chemistry, Columbia University, New York, NY 20017 USA; 3 Present Address: Division of Training, Workforce Development, and Diversity, National Institute of General Medical Sciences, NIH, Bethesda, MD 20892 USA

## Abstract

In concentrated macromolecular solutions, weak physical interactions control the solution behavior including particle size distribution, aggregation, liquid-liquid phase separation, or crystallization. This is central to many fields ranging from colloid chemistry to cell biology and pharmaceutical protein engineering. Unfortunately, it is very difficult to determine macromolecular assembly states and polydispersity at high concentrations in solution, since all motion is coupled through long-range hydrodynamic, electrostatic, steric, and other interactions, and scattering techniques report on the solution structure when average interparticle distances are comparable to macromolecular dimensions. Here we present a sedimentation velocity technique that, for the first time, can resolve macromolecular size distributions at high concentrations, by simultaneously accounting for average mutual hydrodynamic and thermodynamic interactions. It offers high resolution and sensitivity of protein solutions up to 50 mg/ml, extending studies of macromolecular solution state closer to the concentration range of therapeutic formulations, serum, or intracellular conditions.

## Abstract (web summary)

Many aspects of concentrated macromolecular solutions, such as encountered in cytosol or in pharmaceutical formulations, are dependent on particle size distributions and weak intermolecular interactions. Here, the authors exploit hydrodynamic separation in the centrifugal field to measure both.

## Introduction

The physical state of macromolecules at high concentration in solution with regard to their size distribution and physical interactions are of critical importance in many fields such as chemical engineering, food science, colloid chemistry, biochemistry, cell biology, and biotechnology. The state of macromolecules at high concentrations can be governed by a variety of weak interaction mechanisms, for example, weak self-association processes, repulsive steric and hydrodynamic interactions, electrostatic interactions, and depletion forces^1^. The balance of these forces can control an amazingly wide spectrum of behavior, which is well illustrated in our current understanding of cell biology.

With regard to macromolecular interactions, this field has traditionally been dominated by the paradigm of specific and high-affinity interactions between well-structured macromolecules with conserved mutual binding interfaces leading to relatively stable and structurally unique macromolecular complexes. However, the importance of physical, “non-specific” interactions at high intracellular concentrations is increasingly recognized and intensely studied, well beyond the crowding effects on thermodynamics and hydrodynamic interactions controlling intracellular motion^2,3^. For example, weak multi-valent interactions between different (often at least partially intrinsically unstructured) protein species mediated by promiscuous binding interfaces can control the dynamic formation of polymorph multiprotein assemblies associated with diverse cellular functions, such as signaling^4^. Related, liquid–liquid phase separation driven by weak macromolecular interactions in the crowded intracellular environment has recently been recognized as a wide-spread principle of spatial organization, explaining the formation of membrane-less intracellular organelles confining specific cellular activities^5^. These have also been implicated in disease, including some of the many protein aggregation disorders. On the other extreme of protein solution behavior are crystallin proteins in the eye lens. They have evolved to enhance tissue refractive index and are present at extremely high concentrations in excess of 400 mg/ml in the nucleus^6–8^; despite the lack of any cellular metabolic support they are stable for many decades without forming higher-order structures, and they thereby avoid scattering from their aggregation or liquid–liquid phase transition and delay the onset of cataracts.

A similar problem of protein solution state is encountered in a different context of the pharmaceutical industry, where the goal is to engineer stability into protein drug products such that they remain monodisperse over long periods of time at concentrations exceeding 100 mg/ml in therapeutic formulations^9^. An important aspect of protein drug products is the viscosity of the formulation, which is modulated by macromolecular proximity and governed by hydrodynamic and electrostatic forces, as well as weak non-covalent oligomerization processes^10^. Furthermore, the detailed quantitation of oligomeric populations and traces of protein aggregates is essential to assess immunogenicity and to satisfy related regulatory requirements^11,12^. Therefore, understanding both the protein size distribution and weak interactions in solution at high concentrations is indispensable for protein therapeutics and biosimilars in biotechnology^13^, and we emphasize this application in the present work.

Unfortunately, measuring macromolecular size distributions and macromolecular interactions at high concentrations is a formidable experimental challenge. In nonideal solutions the motion of each particle is modulated depending on the position of all others, due to the combined effect of long-range hydrodynamic flow fields, steric and electrostatic forces, pressure effects, solvent- or co-solute-mediated interactions, and solvent back-flow in the finite sample volume^1,14^. This interdependence invalidates the linearity principle underlying current polydispersity analysis in methods relying on stochastic or directed motion, such as dynamic light scattering and sedimentation. Small-angle scattering methods are affected additionally from interference arising from close macromolecular proximity at high concentrations. Although there are several techniques to characterize the solution structure for monodisperse particles at high concentrations, none currently allows to simultaneously resolve the macromolecular size distribution. This limitation is critical since highly concentrated protein samples have the propensity to form higher-order structures such as oligomers, aggregates, or microclusters—structures that in the case of light scattering may dominate the measured signal even at small weight concentrations.

Among the techniques capable of measuring macromolecular size distributions and interactions in solution, analytical ultracentrifugation (AUC) using sedimentation velocity (SV) stands out due to its mass-dependent driving force providing a resolution that surpasses diffusion-based methods^15^. It also features exquisite sensitivity to trace populations, and allows gentle experimental conditions that cause negligible sample dilution, require no tags, and result in minimal surface interactions. SV has become an important technique in pharmaceutical industry^13,16–21^ complementing as an orthogonal method the more high-throughput size-exclusion chromatography by avoiding some potential pitfalls of the latter^21–23^. Besides the study of therapeutic proteins in aqueous buffer systems, SV has been applied to serum and cell lysates^24–29^. Unfortunately, for reasons outlined above, the size distribution analysis of SV breaks down at the onset of solution nonideality. For globular proteins this occurs at concentrations exceeding a few mg/ml, and at much lower concentrations for non-globular macromolecules due to their higher hydrodynamic volume; examples include polymers^30^, unstructured proteins^31^, carbohydrates^32^, mucins^33^, and many polymer-conjugated proteins in biotechnology^30,32,34^.

Nevertheless, as we show here, the mass-dependent separation in SV offers a unique opportunity to measure polydispersity also in nonideal solutions, if nonideality can be accounted for. To this end, we apply a mean-field first-order approximation of nonideality of sedimentation and diffusion, expressed through average interaction parameters *k*_S_ and *k*_D_, incorporated into an extension of the widely used size distribution analysis *c*(*s*). This makes possible a size distribution analysis method termed *c*_NI_(*s*_0_) for polydisperse nonideal systems at high concentrations: It is a diffusion-deconvoluted sedimentation coefficient distribution that is approximately corrected for nonideality, and simultaneously provides estimates for the average nonideality parameters for sedimentation and diffusion. We test this method with experimental data from the highly nonideal solutions of proteins at high concentrations, including suspensions of the NISTmAb reference material, and more polydisperse apoferritin and bovine serum albumin (BSA) samples up to 50 mg/ml. We find *c*_NI_(*s*_0_) can provide excellent fits to nonideal sedimentation data, maintaining excellent resolution and sensitivity at all concentrations tested, and reveal *k*_S_ with excellent precision from a single experiment. This extends high-resolution polydispersity analyses via SV by 1–2 orders of magnitude in concentration, closing the gap between methodological concentration limits and conditions of interest for the study of macromolecules in formulations or intracellular environment.

## Results

### Analysis of nearly monodisperse samples

As a first test to establish that the nonideal sedimentation model can adequately describe the sedimentation process at high concentration, we carried out SV experiments with the NISTmAb standard reference material at different concentrations. Experiments at low concentration of 0.1–0.2 mg/ml were used as a reference point, where the sedimentation data follow very well ideal sedimentation (Supplementary Fig. 1). The best-fit sedimentation coefficient distribution *c*(*s*) shows a monomer peak at *s*_20*w*_ = 6.58 S, translational frictional ratio of 1.6, and reveals the presence of 2.5% dimeric aggregate, the latter consistent with certification of the reference material.

**Fig. 1:**
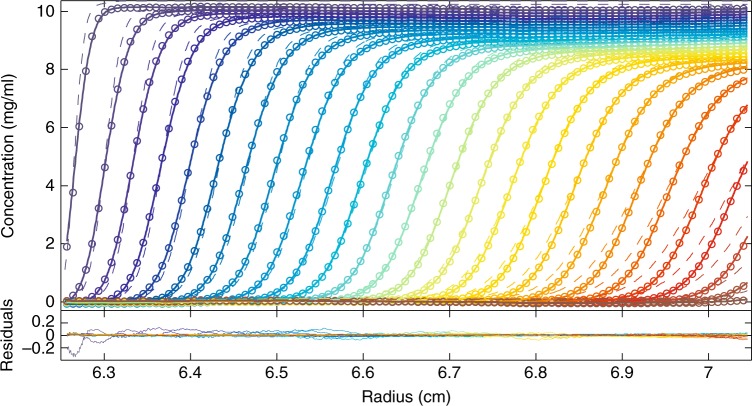
Sedimentation velocity analysis of NISTmAb at 10 mg/ml. Interference data of NISTmAb stock solution in 25 mM histidine buffer, sedimenting at 45,000 rpm in 3 mm centerpieces, are shown with color temperature gradient indicating the boundary migrating from left to right (circles, for clarity only every 10th point is shown). The sedimentation model accounting for nonideality, *c*_NI_(*s*_0_), is shown as solid lines, leading to an rmsd of 0.015 fringes. For comparison, the best-fit standard ideal model *c*(*s*) is shown as dashed lines, leading to an rmsd of 0.098 fringes. In the nonideal model the frictional ratio is fixed to 1.6, whereas in the standard ideal model the frictional ratio is refined to a best-fit value of 3.5. The lower panel shows residuals of the *c*_NI_(*s*_0_) fit

**Fig. 2:**
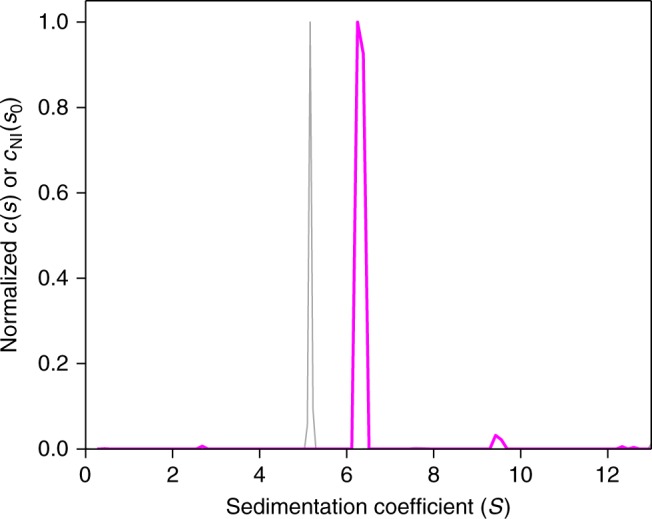
Sedimentation coefficient distribution of NISTmAb. The nonideal *c*_NI_(*s*_0_) model (magenta) associated with the fit shown in Fig. 1 has a monomer peak at 6.56 S, and a dimer peak comprising 2.7% of the material. For comparison, the best-fit standard *c*(*s*) model is shown as thin gray line

As expected, despite the nearly monodisperse size distribution of the NISTmAb, the ideal sedimentation model is unable to describe data acquired at the stock concentration of 10 mg/ml (Fig. 1, symbols and dashed line). Characteristics of nonideal sedimentation boundaries include significant retardation and sustained self-sharpening. The self-sharpening occurs due to the higher sedimentation velocity of molecules in the dilute trailing part of the boundary compared to those in the leading edge of the boundary, where high concentrations with repulsive intermolecular interactions diminish sedimentation. In fact, at very high concentrations this differential creates a migrating boundary with time-independent profile where boundary diffusion is completely counteracted by self-sharpening^35^. A less obvious but also significant deviation from ideal sedimentation is that radial dilution increases the exponential boundary acceleration^15,36^. Therefore, any model assuming ideal sedimentation and diffusion-based boundary broadening cannot describe the nonideal process adequately. It will produce systematic misfits, initially overestimating and later underestimating boundary gradients (dashed lines in Fig. 1). This misfit can serve as a flag to disregard the results, which are characterized by underestimated sedimentation coefficients (gray line in Fig. 2), and grossly overestimated best-fit translational friction coefficients—in the present case amounting to a value of 3.5.

When fitting next the nonideal distribution model *c*_NI_(*s*_0_) to the data, we reduced the number of adjustable parameters by fixing the frictional ratio to the value of 1.6 measured in dilute conditions while refining *k*_S_ and *k*_D_ in curve-fitting. As shown in Fig. 1 (solid lines), this led to an excellent fit of the entire experimental data throughout the sedimentation process. The corresponding best-fit distribution is highly consistent with that measured in dilute conditions, with a monomer peak at 6.56 S, and 2.7% of dimeric aggregate (Fig. 2). The uncertainty of the dimer fraction was assessed from three replicate experiments leading to a standard deviation of 0.8%.

In addition to the sedimentation coefficient distribution, the analysis supplies estimates for interaction parameters. The best-fit estimates of *k*_S_ and *k*_D_ are 19.5 ml/g (19.2–25.5 ml/g, 95% CI) and 45 ml/g (40–56 ml/g, 95% CI), respectively. To assess their validity, we carried out an independent measurement of *k*_S_ from the traditional regression of the weighted-average sedimentation coefficient as a function of loading concentration across a dilution series (Supplementary Fig. 2). This resulted in a value of 21.5 ml/g (19.9–23.1 ml/g, 95% CI), consistent with the value from *c*_NI_(*s*_0_) applied to the stock concentration. We also independently measured *B*_*2*_ by sedimentation equilibrium (SE), arriving at a value of 28 ml/g in the formulation buffer. Based on Eq. (4), we would therefore expect *k*_D_ to be ~35 ml/g, which is in near agreement with the measured *k*_D_-value. Further exploring the information content with regard to *k*_D_, we found that by *c*_NI_(*s*_0_) diffusion nonideality is not very well defined, as indicated by a slight decrease in fit quality when fixing *k*_D_ to 10 ml/g and allowing all other parameters to refine (rmsd 0.022 fringes), in contrast to dramatic changes when applying the same constraint to *k*_S_ (rmsd 0.179 fringes). Interestingly, we also observed the lower *k*_D_-value can be compensated entirely by allowing the frictional ratio parameter to assume an unrealistically low value of 1.3, in a fit associated with false dimer peaks in *c*_NI_(*s*_0_), and a slight increase in *k*_S_ to 25.8 ml/g. This shows a potential correlation between the polydispersity, the frictional ratio (i.e., the scaled ideal diffusion coefficient), and the diffusion interaction parameter. This can be avoided through the initial measurement in dilute conditions, which provides an independent estimate of the frictional ratio to serve as constraint in the *c*_NI_(*s*) analysis. In this way, the distribution and the interaction parameter for sedimentation *k*_S_ can be well defined.

We also carried out an experiment using shorter optical pathlengths in three-dimensional (3D) printed 1.5 mm centerpieces to explore the potential of this strategy for extending the detection range. Again, an excellent fit was achieved with a ratio of rmsd to loading signal of 0.25%, with a consistent *c*_NI_(*s*_0_) distribution exhibiting the monomer peak at 6.66 S, 5.3% dimer and providing best-fit estimates of *k*_S_ and *k*_D_ of 18.4 (17.8–19.0) and 56 (48–63) ml/g, respectively (Supplementary Fig. 3). The reasonable consistency of results and the remarkably good fits of data acquired with either centerpiece excludes Wiener skewing as a significant factor in this concentration range.

**Fig. 3:**
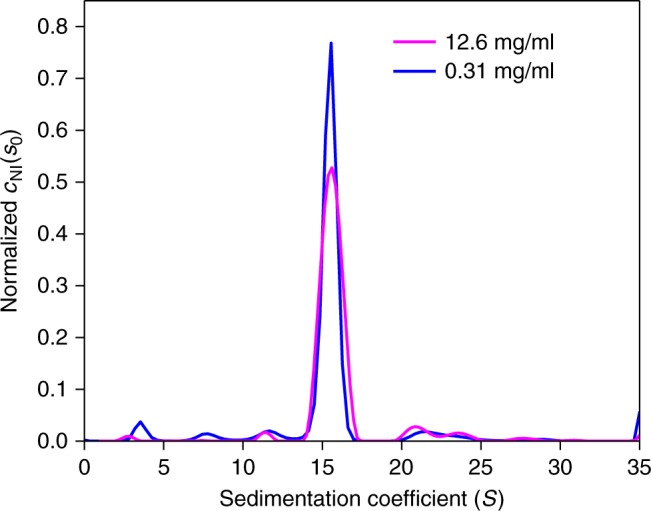
Comparison of sedimentation coefficient distributions of apoferritin samples under ideal and nonideal conditions. Sedimentation data from the nonideal 12.6 mg/ml sample in PBS were analyzed with the nonideal *c*_NI_(*s*_0_) model (magenta). Raw data and fit are shown in Supplementary Fig. 4. The data from a dilute solution of apoferritin at 0.31 mg/ml (blue) were analyzed with the standard ideal *c*(*s*) model

In a second example we examined a more heterogeneous protein sample of apoferritin. Using the same strategy of fixing the diffusion parameter *f*_r_ to the value measured in dilute solution, the data from a 12.6 mg/ml sample could be fit with an rmsd/total signal ratio of 0.11% (Supplementary Fig. 4). The corresponding sedimentation coefficient distribution is highly consistent to that at dilute conditions (Fig. 3), and the nonideality coefficient *k*_S_ of 8.9 (8.5–9.3) ml/g from *c*_NI_(*s*_0_) at 12.6 mg/ml is within error identical to the value of 9.3 (8.9–9.6) ml/g from linear regression based on the *s*-value of the major peak in a concentration series (Supplementary Fig. 5).

### Accounting for Johnston–Ogston distortions in mixtures

**Fig. 4:**
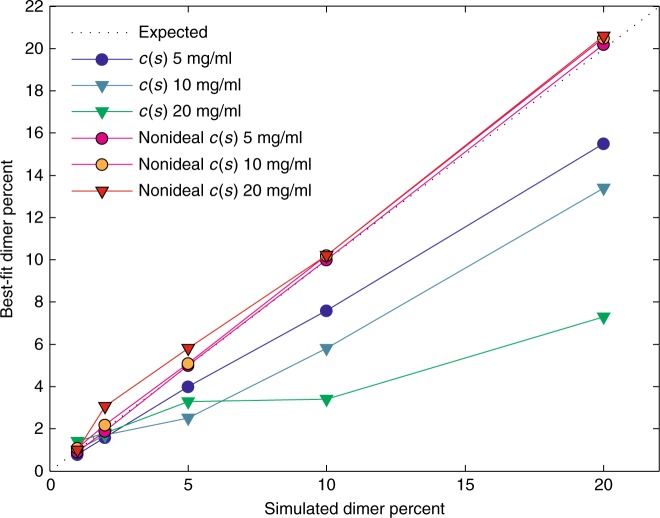
Impact of Johnston–Ogston effect on the detection of dimer populations. Sedimentation data were simulated for an antibody with different dimer populations at 5, 10, and 20 mg/ml total concentrations. Shown are the estimated dimer fractions from analysis using an empirical fit with the standard *c*(*s*) model (blue, cyan, green), and with the *c*_NI_(*s*_0_) model accounting for nonideality (red, orange, and magenta). The true dimer fractions are indicated by the dotted gray line

In comparison to nonideal sedimentation of a single species, multicomponent sedimentation presents an additional, significantly more challenging problem: The same concentration-dependent molecular migration that produces boundary sharpening of single components will for multicomponent mixtures produce boundary anomalies consisting of a concentration maximum for smaller particles trailing the sedimentation boundary of larger particles. Known as the Johnston–Ogston effect (JOE)^37^, this phenomenon is most apparent when the smaller component is specifically detected and a signal maximum in the leading edge of the boundary appears. By contrast, when using non-specific detection, such as interferometry, JOE cannot be visually discerned. Nevertheless, independent of detection JOE creates the same concentration inversion, which appears to magnify the boundary amplitude of the smaller size particles and partially masks the boundary of the faster-sedimenting species. Thus, a naïve measurement of sedimentation boundary amplitudes of different size particles will lead to a skewed distribution underestimating faster-sedimenting populations. To study the impact of this effect on estimating oligomeric protein fractions, we simulated nonideal sedimentation data for an antibody with properties similar to the NISTmAb, but with dimer fractions from 1 to 20% at total concentrations of 5, 10, and 20 mg/ml. Simulations were carried out using a conventional model for two discrete non-ideally sedimenting species with given parameters, and the resulting sedimentation patterns were analyzed with either standard *c*(*s*) or the *c*_NI_(*s*_0_) method allowing for unknown distributions and interaction parameters in the inverse problem. Even though the fit quality of the standard model quickly deteriorates at higher concentrations and reports unreasonable frictional ratios, as mentioned above (Supplementary Tables 1–3), it still appears to allow estimate of oligomeric populations as reflected in boundary amplitudes. However, as shown in Fig. 4, the estimated populations using the standard model are systematically and substantially too low, e.g., reflecting only approximately one-third of the true population at 20 mg/ml—a consequence of JOE. By contrast, the dimer fractions determined from *c*_NI_(*s*_0_) analysis of the same data are close to the true values (Fig. 4). Thus, because *c*_NI_(*s*_0_) is designed to model the mutual nonideality interaction between different species, the JOE is naturally accounted for and size distributions are unskewed.

**Fig. 5:**
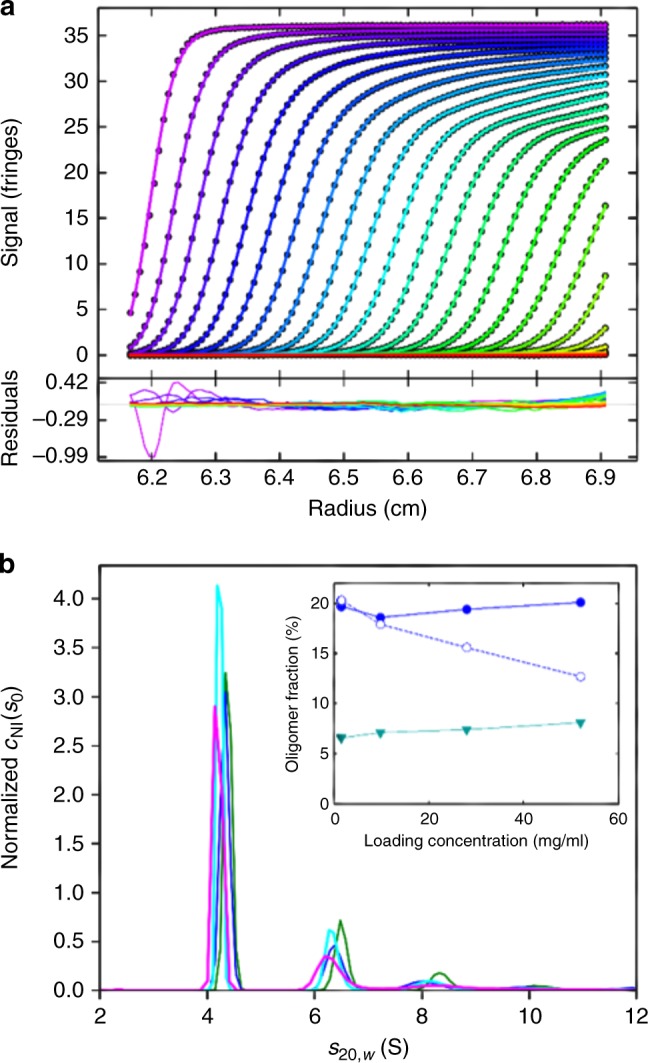
Sedimentation analysis of BSA at 52 mg/ml in PBS. **a** Interference optical data were acquired in a 3 mm centerpiece (circles, showing every 10th data point of every third scan). The best-fit *c*_NI_(*s*_0_) model (solid lines) with a fixed frictional ratio of 1.45 results in an rmsd of 0.058 fringes, with *k*_S_ = 8.4 ml/g and *k*_D_ = 10 ml/g. **b** The associated sedimentation coefficient distribution at 52 mg/ml (magenta) and, for comparison, the distributions obtained in analogous analyses at 28 mg/ml (cyan), 9.8 mg/ml (blue), both also acquired in 3 mm centerpieces, and 1.6 mg/ml (green) acquired in a 12 mm centerpiece. The inset shows integrated dimer fractions at ~6.5 S (solid blue circles) and trimer fractions at ~8 S (solid green triangles) from these *c*_NI_(*s*_0_) analysis accounting for nonideality. The open circles are the apparent dimer fractions from empirical *c*(*s*) analyses of the same data neglecting nonideality contributions (Supplementary Fig. 7)

In order to demonstrate this effect experimentally, we carried out SV experiments with BSA at concentrations from 0.4 to 52 mg/ml. Without further purification, BSA is well known to exhibit significant populations of stable oligomeric species such as dimers and trimers. As shown in Fig. 5a, even at the highest concentration of 52 mg/ml the *c*_NI_(*s*_0_) model described the sedimentation data remarkably well, with an rmsd corresponding to only 0.16% of the loading signal. The estimate of *k*_S_ of 8.4 ml/g derived from the fit in Fig. 5 is consistent with previously published data from concentration series^38^. The sedimentation coefficient distribution provides baseline-resolved monomer, dimer, and trimer populations, demonstrating that the deconvolution of diffusional boundary broadening works in the nonideal case similar to the analysis of ideal solutions (Fig. 5b). Finally, the oligomer fractions are consistent across the entire concentration series and with those in dilute solution, which demonstrates that boundary anomalies from JOE in highly concentrated samples are overcome in the experimental analysis with *c*_NI_(*s*_0_) (Fig. 5b, inset). By contrast, the ideal model *c*(*s*) is unable to model the sedimentation process well (Supplementary Fig. 6), and—to the extent that the resulting apparent distribution can be considered meaningful—shows declining apparent dimer fractions (Fig. 5b inset, open circles) that are a direct result of JOE.

## Discussion

In the present work we have developed a method extending the measurement of macromolecular size distributions to high concentrations. It is based on SV, a widely used method that exploits the strongly size-dependent migration of particles in the centrifugal field in a configuration that avoids sample sequestration and leads to minimal dilution. We have introduced a computational data analysis framework that can account for mutual hydrodynamic interactions at the onset of nonideality, overcoming the nonlinearity problem intrinsic to the analysis of macromolecular size distributions under crowded conditions where macromolecular motions are correlated. Measurement at high concentration provides a luxurious signal/noise ratio; thus the exceptionally good fits we obtained up to 52 mg/ml show both that the mean-field approximations in the model still capture the salient aspects of the sedimentation process, including the boundary anomalies characteristic for SV at high concentrations, and that the experimental detection does not suffer from significant artifacts. We found that the resolution, sensitivity, and performance of the method for nonideal solutions is comparable to the widely used *c*(*s*) analysis for ideal solutions.

This opens a unique window to study macromolecular polydispersity in solution under nonideal conditions, which may occur not only at high concentrations in excess of 1 mg/ml, but already at more moderate concentrations for extended polymers or unstructured proteins. Besides the size distribution in solution, the method provides a precise estimate of the nonideality coefficient *k*_S_. This parameter is closely related to the second virial coefficient, which has attracted interest in the search for solution conditions for crystallization^39^ and for pharmaceutical formulations^9,10^. The possibility to obtain *k*_S_ from a single experiment will facilitate the application of SV to screen solution conditions, simultaneous to the determination of protein size distribution.

Measurements at high concentration have been a long-standing goal in AUC, for example, in view of the impact of macromolecular crowding in cytosol and serum, in addition to the interest in pharmacological formulations^3,24–28^. Previous experimental work to extend the concentration range of SV included an improved imaging systems to record steeper fringe profiles^40^, short pathlength centerpieces^40,41^, and confocal fluorescence detection to minimize optical skewing^29^. While these tools likely become critical when exploring the methodological limits, we were able to carry out experiments at ~50 mg/ml in standard commercial equipment using a ProteomeLab AUC applying interference optics. 3D printed centerpieces with shorter pathlength, as applied in the present work for comparison, have the potential to further increase the dynamic range. However, the dimer fraction of 5.3% measured for the NISTmAb using the 3D printed centerpieces is slightly elevated compared to the value of 2.7% obtained from samples in commercial Epon 3 mm centerpieces. This may possibly be due to imperfections in the 3D printed centerpieces causing convective disturbances, reminiscent of elevated dimer fractions observed in earlier, less precisely manufactured batches of commercial Epon centerpieces^42^. Optimized experimental strategies and concentration limits will be further explored elsewhere.

The most critical previous limitation has been the failure of standard approaches to correctly interpret SV experiments under nonideal conditions. Boundary modeling suffers not only from the retardation of sedimentation and the implausibly small apparent diffusion coefficients (or high frictional ratios) from boundary sharpening, but, most importantly, from modulation of boundary amplitudes as a result of the Johnston–Ogston effect. Thus, as we have demonstrated here, even analyses that attempt only to extract minimal information on the populations associated with approximate *s*-values will suffer from the systematic underestimate of faster-sedimenting populations. For example, this will further exacerbate distortions in the *dcdt*-method to determine an apparent sedimentation coefficient distribution *g*(s*)^43^, rendering any quantitative interpretation of *g*(s*) uncertain.

Previous methods to more rigorously analyze nonideal SV were limited to discrete models with one or a few species^44^, and often could only be applied to data subsets from a small time window^45,46^, even then usually exhibiting comparatively poor fit qualities. We believe the need for a priori knowledge of the number of species present can be particularly problematic at high concentrations where many proteins exhibit some degree of aggregation. By contrast, the *c*_NI_(*s*_0_) method introduced here provides a physically sensible model for unknown mixtures of sedimenting particles with nonideal interactions, and can describe well the entire sedimentation process.

One theoretical limitation of the model is the mean-field approximation that reduces the mutual nonideality coefficients to a single set of average coefficient *k*_S_ and *k*_D_. The most important parameter in modeling SV is *k*_S_^44,47^. The dominating hydrodynamic contribution to *k*_S_ is dependent not on size but on shape, specifically the cube power of the friction ratio^14,48^. In this way, the description of the entire distribution with a single *k*_S_-value is closely related to the reduction of molecular diffusion constants to a single, average frictional ratio *f*_r_ as a scaling parameter for the distribution. This does not always apply, but is a time-honored simplification that usually matches well the information content of SV data sets (as many applications of *c*(*s*) in the ideal case show)^14,49^, especially when working with samples that are not too polydisperse and of similar class of hydrodynamic shape^50^. With regard to *k*_D_, we found this parameter to be much less well determined, and conversely we can conclude that approximations in this parameter will not impact the model very much, especially for minority components.

If the diffusion parameter *k*_D_ is of interest, one might expect that SV experiments at higher concentrations be helpful in that they generate more information on *k*_D_. However, boundary sharpening governed by *k*_S_ will increasingly dominate boundary shapes and mask diffusional spread, and in the extreme case generate time-independent boundary shapes^35,51^. Potential future extensions of *c*_NI_(*s*_0_) to higher concentrations might reveal whether this leads to an increase or further reduction in information content regarding the distinction between *k*_D_ and polydispersity. Indirect determination of *k*_D_ via *k*_S_ and *B*_2_, exploiting other techniques such as SE or light scattering to measure the virial coefficient may be more promising. In this regard, the information on sample homogeneity from the *c*_NI_(*s*_0_) analysis of SV experiments should be useful beyond providing a *k*_S_-value.

Another limitation intrinsic to the current method is the need to record signals that reflect weight concentrations of all species equally. Due to the narrow range of macromolecular refractive index increments, this is fulfilled in good approximation when using Rayleigh interference optics. If only selective signals are available, as in fluorescence-detected SV, the difficulty arises that the majority of sedimenting particles—the detailed sedimentation patterns of which locally control nonideality—may remain invisible. This limits the study to single-component solutions. For example, it renders very problematic the attempt to measure nonideal cross-coefficients of different species from data based on selective detection of only one species^45^. A possible solution could be the creation of signal boundaries at radial positions in solution zones without concentration gradients (in the plateau regions of all species) using photoswitchable tags and structured illumination techniques introduced recently to SV^52^. This would also considerably simplify analysis by eliminating boundary sharpening and JOE effects. Alternatively, the extension of the *c*_NI_(*s*_0_) method to multi-signal SV^53^ should be seamless, which would open the door to study more complex multicomponent systems exhibiting hetero-associations.

## Methods

### Nonideal sedimentation coefficient distributions

We describe in the following the mathematical structure of the sedimentation coefficient distributions *c*_NI_(*s*_0_), then summarize the foundation of sedimentation and diffusion in nonideal solutions, and finally show how *c*_NI_(*s*_0_) and the nonideality parameters are computationally coupled and calculated.

The basic structure of the model follows the *c*(*s*) distribution of ideal particles^54^. The goal is to calculate a sedimentation coefficient distribution *c*(*s*), reflecting the concentration *c* of species with sedimentation coefficients between *s* and *s* *+* d*s*, from experimental signals *a*(*r*,*t*) observed as a function of radius *r* and time *t*, by least-squares fitting a linear superposition of signals of species with sedimentation coefficient *s* and diffusion coefficient *D*,

1 $$a\left( {r,t} \right) \cong \varepsilon d\mathop {\int}\nolimits_{\hskip -5pts_{\min }}^{s_{\max }} {c\left( s \right)\chi \left( {s,D\left( {f_{\mathrm{r}},s} \right),r,t} \right){\mathrm d}s}$$

where *ε* is an extinction coefficient and *d* the optical pathlength, and *χ*(*r*,*t*) denotes the species spatio-temporal sedimentation pattern at unit concentration. Utilizing the time-honored approximation that *s* scales with molar mass *M* as *s~M*^2/3^ for globular particles, as in the majority of *c*(*s*) applications, the diffusion coefficient is estimated from the sedimentation coefficient on the basis of a hydrodynamic scaling law for an ensemble of globular particles characterized by a common translational frictional ratio *f*_*r*_. (However, it should be noted that *c*_NI_(*s*_0_) will be naturally compatible with other hydrodynamic scaling laws, including those for random chains^54,55^.) *a*(*r*,*t*) should comprise all available scans throughout the sedimentation process, such that evolution of the sedimentation boundaries can provide information on polydispersity and diffusion, in addition to nonideality. In this way the residuals offer a stringent criterion for the validity of the model. Additional computational aspects added to Eq. (1) arising from maximum entropy or Tikhonov regularization, as well as those related to noise decomposition, follow standard procedures^54^ and are suppressed here for clarity.

For numerical solution of Eq. (1) a grid of *N* species *i* = 1*…N* is considered. The calculation of each species’ sedimentation patterns *χ*_*i*_(*r*,*t*) harbors the key difficulty of nonideality. In the limit of ideal sedimentation *χ*_*i*_(*r*,*t*) follows the Lamm partial differential equation^56^

2 $$\frac{{\partial \chi _i}}{{\partial t}} = - \frac{1}{r}\frac{\partial }{{\partial r}}\left( {s_i\omega ^2r^2\chi _i - D_i\frac{{\partial \chi _i}}{{\partial r}}} \right)$$

with constant *s*_*i*_ and *D*_*i*_, which can be solved using numerical principles described previously^54^. Eq. (2) may also involve time-dependent rotor speeds *ω*(*t*) as in gravitational sweep sedimentation^57^. In nonideal sedimentation the same transport Eq. (2) is valid, but sedimentation and diffusion coefficients now become dependent on the local total macromolecular concentration *χ*_tot_(*r*,*t*),

3 $$\begin{array}{l}s_i\left( {r,t} \right) = s_{0,i}\left( {1 - k_{\mathrm{S}}\chi _{\mathrm {tot}}\left( {r,t} \right)} \right) = s_{0,i}\left( {1 - k_{\mathrm{S}}{\int} {c\left( s \right)\chi _j\left( {s,r,t} \right){\mathrm d}s} } \right)\\ D_i\left( {r,t} \right) = D_{0,i}\left( {1 + k_{\mathrm{D}}\chi _{\mathrm {tot}}\left( {r,t} \right)} \right) = D_{0,i}\left( {1 + k_{\mathrm{D}}{\int} {c\left( s \right)\chi _j\left( {s,r,t} \right){\mathrm d}s} } \right)\end{array}$$

with parameters at infinite dilution denoted by subscript “0”, and with interaction parameters (synonymously termed nonideality coefficients) for sedimentation *k*_S_ and for diffusion *k*_D_ expressing a first-order approximation for the concentration dependence. Equation (3) makes a mean-field approximation that mutual nonideality coefficients of different species can be described by a single average coefficient *k*_S_, and analogous for *k*_D_.

The nonideality coefficients are related by hydrodynamic theory of a single class of particles^44,47,58^ to the second virial coefficient *B*_2_, which can be written as

4 $$2B_2 = k_{\mathrm S} + k_{\mathrm D}$$

and is a function of the macromolecular distance distribution and the interparticle potential *W*(*r*)

5 $$B_2 = 2\pi {\int} {\left( {1 - {\mathrm e}^{ - W\left( r \right)/kT}} \right)r^2{\mathrm d}r}$$

for quasi-spherical particles^59^. Furthermore, Batchelor has shown that

6 $$s = s^0\left( {1 - \left[ {6.55 - 3.53\left( {1 - B_2/B_{{\mathrm{HS}}}} \right)} \right] {\Phi} } \right),$$

where *B*_HS_ is the hard sphere virial coefficient and *Φ* is the volume fraction occupied by macromolecules^60^, establishing a relationship between *k*_S_, the molecular distance distribution, *W(r)*, and *B*_2_.

The central difficulty of solving the integral equation Eq. (1) in the nonideal case is that the transport coefficients in Eq. (3) are dependent already on the integral in Eq. (1). This prohibits the application of previously used standard methods for solving Fredholm integral equations of the first kind^54^, since Eq. (1) has now become an implicitly defined nonlinear integro-differential equation for *c*(*s*) that is considerably harder to solve. In the present work, we have developed an iterative procedure: Initially *χ*_tot_(*r*,*t*) is approximated across the time-course of the experiment to solve Eq. (3). This approximation is used to calculate sedimentation patterns for each species via Eq. (2), which is followed by a standard linear decomposition of the integral equation in Eq. (1). The latter yields a better approximation of *χ*_tot_(*r*,*t*) to repeat these steps. After suitable initialization, the second iteration for solving Eq. (1) provides only a small improvement, but further iterations are folded into the optimization of nonlinear adjustable parameters including *k*_S_, *k*_D_, the solution meniscus, and optionally *f*_r_ and baseline signals. Similarly, signal offsets from sedimenting co-solutes resulting from buffer composition mis-matches between sample and reference sectors can be included in the fit. (Modeling the impact on macromolecular sedimentation of dynamic density and viscosity gradients at high co-solute concentrations^61,62^ is compatible with the current model but not yet implemented; this limits the application at present to sufficiently dilute buffer conditions.) We have implemented this algorithm in SEDFIT as multi-threaded Windows executable achieving run-times of seconds on conventional laptop computers in applications to full experimental data sets of 50–100 scans with a number of 100–200 *s*-values serving as grid points in the sedimentation coefficient distribution.

The nonideal sedimentation coefficient distribution based on Eqs. (1)–(3) is designated *c*_NI_(*s*_0_), the subscript “0” reflecting the fact that it will represent sedimentation coefficients in units at infinite dilution of the macromolecules at experimental solvent conditions. For structural and other absolute interpretations of sedimentation coefficients, buoyancy and viscosity corrections may be applied to *s*-values of the final *c*_NI_(*s*_0_) distributions—if partial-specific volume data are available—so as to standardize *s*-value units to water at 20 °C^15^. Since nonideality coefficients in Eq. (3) describe fractional reduction of *s*-values, they are independent of *s*-value units. This is in contrast to an approach where *k*_S_ is calculated by a linear regression of standardized *s*-values: Here, buoyancy corrections may be calculated with solution density or with solvent density, viewpoints that are ultimately equivalent but result in *k*_S_-values that differ by an amount equaling the partial-specific volume^44,47,48,58^. The *k*_S_-values from *c*_NI_(*s*_0_) will naturally correspond to the solvent-density corrected framework, consistent with the hydrodynamic theory of Batchelor^63,64^.

### Analytical ultracentrifugation

AUC experiments were carried out with a ProteomeLab instrument (Beckman Coulter, Indianapolis) following standard methods^15^. Briefly, calibration correction factors for the instrument with regard to time, radius, and temperature were determined as previously described^65–67^, cumulatively amounting to a factor 1.015. NISTmAb (SRM 8671, NIST, Gaithersburg) was diluted in 25 mM l-histidine buffer, pH 6.0; BSA and apoferritin (Sigma, St. Louis, cat #A7030 and A3641) were resuspended in phosphate-buffered saline pH 7.4 (PBS). Unless otherwise mentioned, samples were filled in 3 mm or 12 pathlength Epon double sector centerpieces. For SV experiments, the sample volume was chosen to create 12 mm long solution columns. After assembly, the AUC cells were placed in an 8-hole rotor, and temperature equilibrated at 19.8 °C while resting in vacuum in the rotor chamber. Acceleration to 26,000 rpm for apoferritin, 45,000 rpm for NISTmAb, or 50,000 rpm for BSA, respectively, was immediately followed by acquisition of radial scans using the Rayleigh interference optical system. SE experiments were carried out with 6 mm long solution columns, using time-optimized rotor speed profiles^68^ to attain SE at 9,000 rpm. SE data were globally modeled in SEDPHAT using the INVEQ method by Ang and Rowe^69^ to account for nonideality with the second virial coefficient.

At high concentrations the data quality will be dominated by systematic detection errors. The Rayleigh interference optical detection provides the largest dynamic range but the fringe shift assignment of the Beckman ultracentrifuges fails if the fringe shift from neighboring camera pixels exceeds half a fringe, which occurs at gradients above 70 fringes/mm. This results in discontinuities of the radial fringe profile^70^. To some extent this may be corrected by data pre-processing restoring continuity of derivatives in the signal trace, as implemented in SEDFIT^70^. A second limitation occurs from high refractive index gradients causing an optical aberration known as Wiener skewing, which will distort the measured concentration gradients. Svensson has shown to that the magnitude of skewness is proportional to *d*^3^ × (d*n*/d*r*) ^2^ × (2/3−*a*), where *d* is the optical pathlength, d*n*/d*r* is the refractive index gradient, and *a* is the focal depth relative to the sample height^71^. Using a focal depth in the AUC instrument at the 2/3 plane of the optical pathlength will eliminate this distortion, but it is impractical to adjust for most users. However, the cube dependence on total pathlength makes skewing much less problematic for short pathlength cells, and provides a convenient approach to virtually eliminate this problem. For example, 3 mm cells will lead to 4-fold reduced signal and 64-fold reduced skew. We have previously shown experimentally that even with errors in the focal point up to 1.5 mm no detectable skew occurs at gradients of 10 fringes/cm, such as generated with ≈20 mg/ml BSA samples in 3 mm pathlength cells at 50,000 rpm^70^.

Thus, to experimentally minimize both fringe skipping and optical skewing, we have designed centerpieces with 1.5 mm pathlength, which extend the dynamic range by a factor of two and reduce skew by a factor of four relative to 3 mm cells. We have previously introduced 3D printing of AUC centerpieces and shown their excellent performance when printed in various materials, including Microfine Green used in the present work (Protolabs, Maple Plain, MN)^72^. We further improved this design by adding an embossed rim of 100–150 µm height on both top and bottom of the centerpiece to serve as a gasket, and added venting holes to facilitate sample loading. Design files can be downloaded from 3dprint.nih.gov (model ID 3DPX-009261).

For statistical analysis, unless mentioned otherwise, F-statistics was used to determine the root-mean-square deviation (rmsd) related to the 95% confidence level contour of the error surface encompassing the parameters range in this confidence interval (CI). Plots were created using GUSSI^73^ or MATLAB (MathWorks, Natick, MA).

## Electronic supplementary material

Supplementary Information

Peer Review File

## Data Availability

The data that support the findings of this study are available from the corresponding author upon reasonable request. C++ code is compiled as part of the SEDFIT platform, freely available as multi-threaded Windows executable for download from sedfitsedphat.nibib.nih.gov/software.
