# Supplementary material for: Measuring macromolecular size distributions and interactions at high concentrations by sedimentation velocity
Source: Nat Commun. 2018 Oct 24;9:4415. doi: 10.1038/s41467-018-06902-x (PMC6200768; doi:10.1038/s41467-018-06902-x)
Supplement: Supplementary file 1 — Supplementary Information [file 41467_2018_6902_MOESM1_ESM.pdf]

Supporting Information for:

Measuring Macromolecular Size-Distributions and Interactions at High Concentrations by Sedimentation Velocity

Chaturvedi et al

Supplementary Figures 1-7

Supplementary Tables 1-3

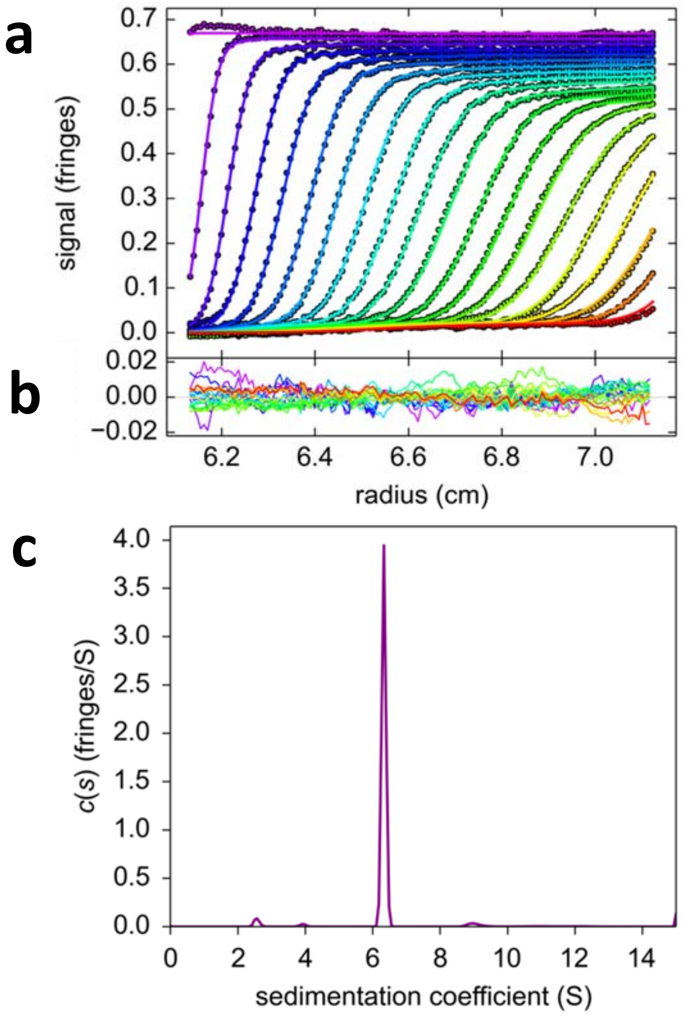

Supplementary Figure 1. Sedimentation velocity analysis of NISTmAb in dilute solution. (a) Interference data acquired during sedimentation of NISTmAb at 0.2 mg/ml at 45,000 rpm in a standard 12 mm centerpiece. For clarity, circles show only every 10th data point of every 3rd scan. The standard $c(s)$ sedimentation coefficient distribution analysis results in a fit indicated by solid lines, using color temperature gradient to indicate the time-points of the boundary migrating from left to right. (b) Residuals of the fit, with a root-mean-square deviation (rmsd) of 0.0045 fringes. (c) Resulting sedimentation coefficient distribution, exhibiting a main peak at 6.33 S (uncorrected s-values; s_{20w} = 6.58 S after calibration corrections) and a dimer peak at 9.04 S comprising 2.5% of the total material.

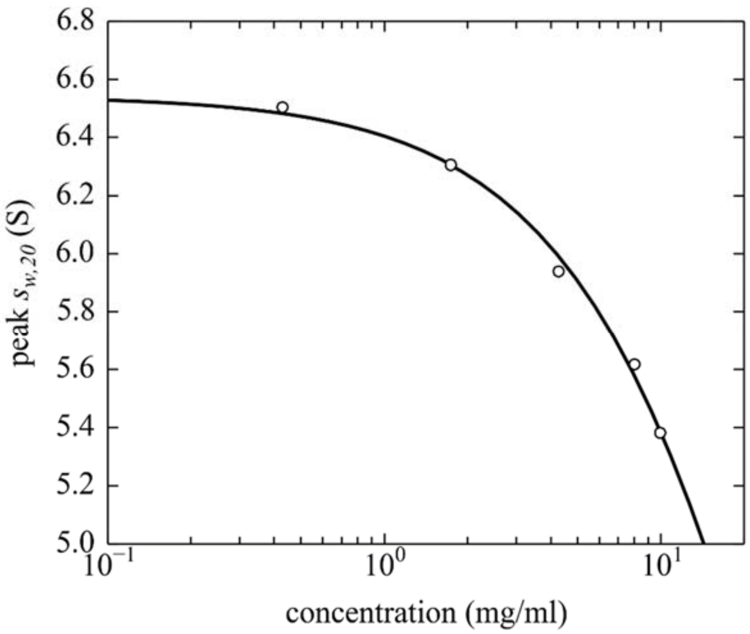

Supplementary Figure 2. Determination of the nonideality coefficient k_S of the NISTmAb from concentration series. Shown is the regression of monomer peak *s*-values from standard *c*(*s*) analysis observed as a function of concentration (see Eq. 3). Concentration values are loading concentrations corrected for the average radial dilution. The best-fit value is 21.5 ml/g (19.9 – 23.1 ml/g, 95% CI, determined by Monte-Carlo analysis).

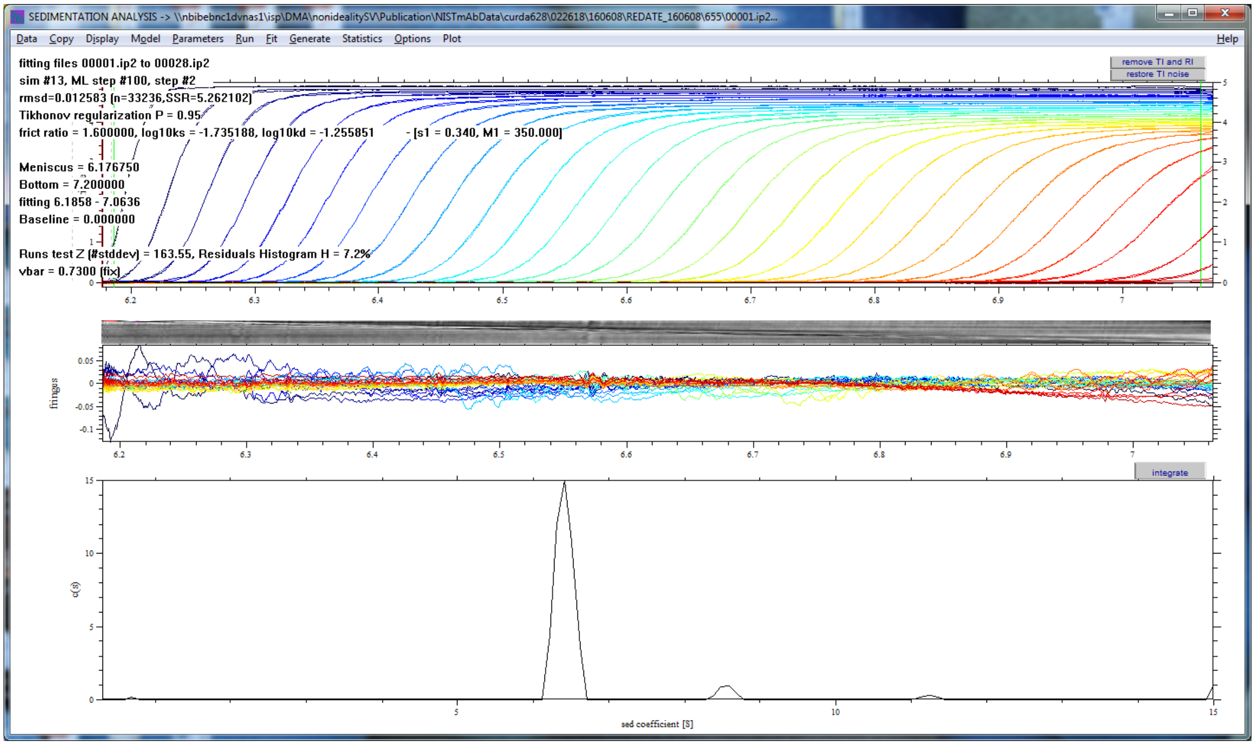

Supplementary Figure 3. Sedimentation analysis of NISTmAb at high concentration in short pathlength 3D printed centerpiece. Shown is a screenshot of the analysis of NISTmAb at 10 mg/ml sedimenting at 45,000 rpm in a custom 1.5 mm centerpiece. Data were fitted with the nonideal $c_{NI}(s_0)$ model, resulting in an rmsd of 0.0125 fringes, and sedimentation coefficient distribution exhibiting the monomer peak at 6.66 S (after calibration correction), with 5.3% of material in a dimer peak, and best-fit estimates of k_S and k_D of 18.4 and 55.5 ml/g, respectively.

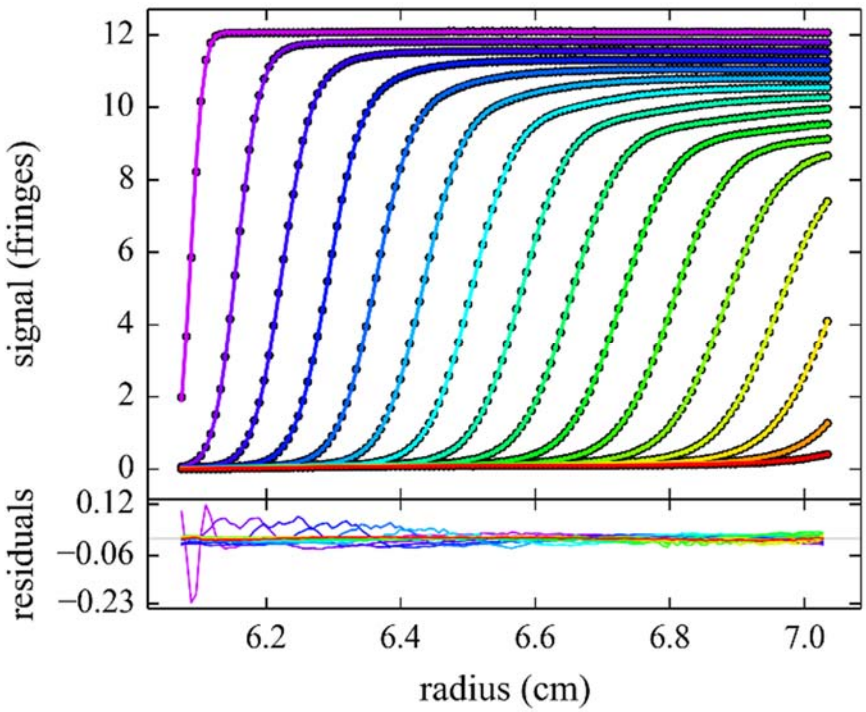

Supplementary Figure 4. Sedimentation analysis of apoferritin at high concentration. Concentration profiles of apoferritin at 12.6 mg/ml in PBS were acquired with the interference optics during sedimentation at 26,000 rpm in a 3 mm centerpiece (circles; only every 10th data point of every 3rd scan shown). The solid lines are the fit with the nonideal $c_{NI}(s_0)$ model, resulting in an rmsd of 0.0129 fringes. The associated sedimentation coefficient distribution is shown in **Fig. 3**.

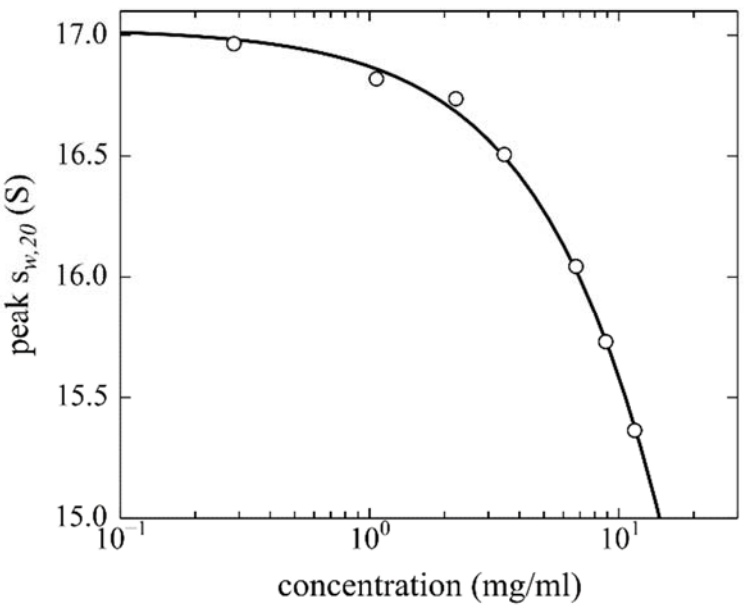

Supplementary Figure 5. Determination of the nonideality coefficient k_S of apoferritin in PBS through concentration series. Data points and solid line show the regression of monomer peak *s*-values in standard *c*(*s*) analysis observed as a function of concentration (see Eq. 3). Concentration values are loading concentrations corrected for the average radial dilution. The best-fit value is 9.3 ml/g (8.9 – 9.6 ml/g, 95% CI, determined by Monte-Carlo analysis).

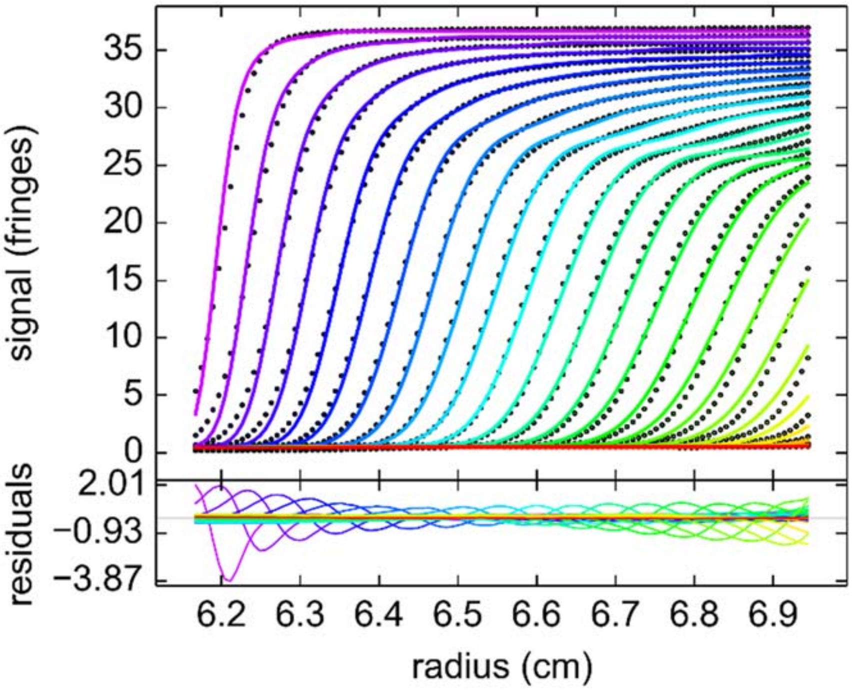

Supplementary Figure 6. Impostor model of BSA sedimentation data at 52 mg/ml with a standard *c*(*s*) model. Sedimentation data are the same as shown in **Fig. 5** (circles; only every 10th data point of every 3rd scan shown). The solid lines are the best-fit standard *c*(*s*) model for ideal solutions, the neglect of nonideality resulting in large systematic deviations.

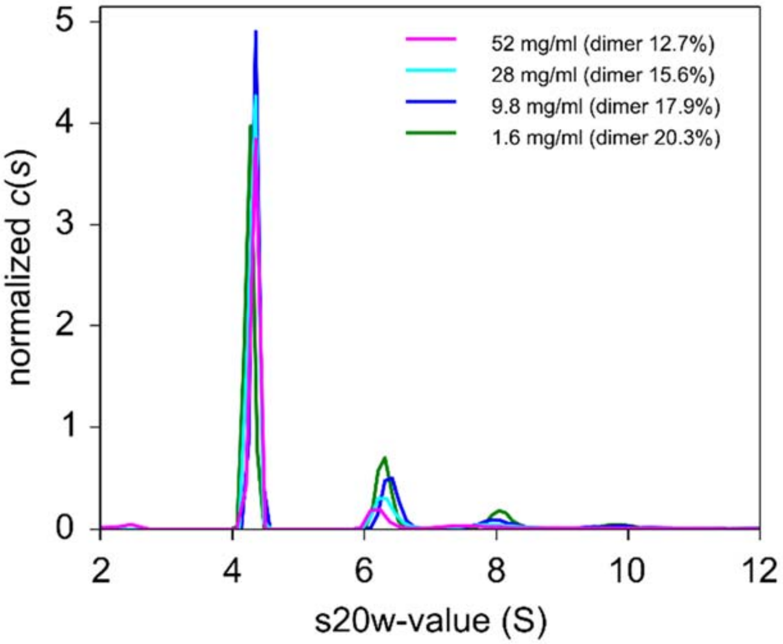

Supplementary Figure 7. Imposter *c*(*s*) distributions modeled to BSA at different concentrations. Shown are results from BSA at 52 mg/ml (magenta), 28 mg/ml (cyan), 9.8 mg/ml (blue), and 1.6 mg/ml (green). Data were acquired by interference optics using 3 mm centerpieces (52 mg/ml, 28 mg/ml, and 9.8 mg/ml) or 12 mm centerpieces (1.6 mg/ml), respectively. The normalized *c*(*s*) distributions exhibit apparent dimer fractions that decrease with increasing concentration, as a result of the Johnston-Ogston effect.

Supplementary Table 1: Impact of Johnston-Ogston distortions at 5 mg/ml protein

Nonideal sedimentation data were simulated for an antibody with properties similar to the NISTmAb, but with dimer fractions from 1 % to 20% at total concentration of 5 mg/ml. Simulations were carried out using a sedimentation model for two non-ideal discrete species. The resulting sedimentation patterns were analyzed with standard $c(s)$ and $c_{NI}(s_0)$. The tables present the rmsd of these models, the peak s-values in the associated distributions, best-fit (or fixed) frictional ratios, and the apparent dimer fractions from integration of the dimer peaks of the distributions. In some conditions additional ghost peaks occur, as indicated in parenthesis.

simulation 5mg/ml	parameters	standard c(s) distribution		nonideal c(s)	
		**s value**	**% abundance**	**s value**	**% abundance**
99% monomer (6.33 S) + 1 % dimer (9.41 S)	**peaks**	5.82<br>8.92	98.8<br>0.8	6.34<br>9.56	99.0<br>0.9
	**f/f0**<br>**rmsd**	2.5<br>0.027		1.6 (fix)<br>0.010	
98% monomer (6.33 S) + 2 % dimer (9.41 S)	**peaks**	5.82<br>8.78	98.0<br>1.6	6.34<br>9.50	98.0<br>1.9
	**f/f0**<br>**rmsd**	2.5<br>0.026		1.6 (fix)<br>0.010	
98% monomer (6.33 S) + 5 % dimer (9.41 S)	**peaks**	5.84<br>8.70	95.6<br>4.0	6.34<br>9.45	95.0<br>5.0
	**f/f0**<br>**rmsd**	2.5<br>0.025		1.6 (fix)<br>0.010	
90% monomer (6.33 S) + 10 % dimer (9.41 S)	**peaks**	5.86<br>8.59	91.2<br>7.6	6.30<br>9.38	90.0<br>10.0
	**f/f0**<br>**rmsd**	2.4<br>0.022		1.6 (fix)<br>0.010	
80% monomer (6.33 S) + 20 % dimer (9.41 S)	**peaks**	5.90<br>8.60	82.7<br>15.5	6.32<br>9.41	79.8<br>20.2
	**f/f0**<br>**rmsd**	2.3<br>0.019		1.6 (fix)<br>0.010	

Supplementary Table 2: Impact of Johnston-Ogston distortions at 10 mg/ml protein

Nonideal sedimentation data were simulated for an antibody with properties similar to the NISTmAb, but with dimer fractions from 1 % to 20% at total concentration of 10 mg/ml. Simulations were carried out using a sedimentation model for two non-ideal discrete species. The resulting sedimentation patterns were analyzed with standard $c(s)$ and $c_{NI}(s_0)$. The tables present the rmsd of these models, the peak s-values in the associated distributions, best-fit (or fixed) frictional ratios, and the apparent dimer fractions from integration of the dimer peaks of the distributions. In some conditions additional ghost peaks occur, as indicated in parenthesis.

simulation 10 mg/ml	parameters	standard c(s) distribution		nonideal c(s)	
		**s value**	**% abundance**	**s value**	**% abundance**
99% monomer (6.33 S) +<br>1 % dimer (9.41 S)	**peaks**	5.31<br>8.09	98.2<br>1.0	6.31<br>9.24	98.8<br>1.1
	**f/f0**<br>**rmsd**	4.3<br>0.098		1.6 (fix)<br>0.012	
98% monomer (6.33 S) +<br>2 % dimer (9.41 S)	**peaks**	5.32<br>8.03	97.5<br>1.7	6.29<br>(8.41)<br>9.36	97.5<br>0.5<br>1.7
	**f/f0**<br>**rmsd**	4.2<br>0.097		1.6 (fix)<br>0.013	
95% monomer (6.33 S) +<br>5 % dimer (9.41 S)	**peaks**	5.34<br>7.67	95.5<br>2.5	6.32<br>9.41	94.7<br>5.1
	**f/f0**<br>**rmsd**	4.2<br>0.092		1.6 (fix)<br>0.011	
90% monomer (6.33 S) +<br>10 % dimer (9.41 S)	**peaks**	5.38<br>7.78	92.2<br>5.8	6.30<br>(7.78)<br>9.37	89.6<br>0.2<br>10.0
	**f/f0**<br>**rmsd**	3.9<br>0.085		1.6 (fix)<br>0.013	
80% monomer (6.33 S) +<br>20 % dimer (9.41 S)	**peaks**	5.45<br>7.82	85.0<br>13.4	6.37<br>9.49	79.3<br>20.5
	**f/f0**<br>**rmsd**	3.6<br>0.070		1.6 (fix)<br>0.011	

Supplementary Table 1: Impact of Johnston-Ogston distortions at 20 mg/ml protein

Nonideal sedimentation data were simulated for an antibody with properties similar to the NISTmAb, but with dimer fractions from 1 % to 20% at total concentration of 20 mg/ml. Simulations were carried out using a sedimentation model for two non-ideal discrete species. The resulting sedimentation patterns were analyzed with standard $c(s)$ and $c_{NI}(s_0)$. The tables present the rmsd of these models, the peak s-values in the associated distributions, best-fit (or fixed) frictional ratios, and the apparent dimer fractions from integration of the dimer peaks of the distributions. In some conditions additional ghost peaks occur, as indicated in parenthesis.

simulation 20 mg/ml	parameters	standard c(s) distribution		nonideal c(s)	
		**s value**	**% abundance**	**s value**	**% abundance**
99% monomer (6.33 S) +<br>1 % dimer (9.41 S)	**peaks**	4.29<br>6.81	95.8<br>1.4	6.36<br>(7.78)<br>9.65	98.5<br>0.5<br>1.0
	**f/f0**<br>**rmsd**	10.9<br>0.397		1.6 (fix)<br>0.024	
98% monomer (6.33 S) +<br>2 % dimer (9.41 S)	**peaks**	4.30<br>6.77	95.4<br>1.8	6.29<br>(7.91)<br>9.76	96.6<br>1.8<br>1.25
	**f/f0**<br>**rmsd**	10.8<br>0.394		1.6 (fix)<br>0.034	
98% monomer (6.33 S) +<br>5 % dimer (9.41 S)	**peaks**	4.34<br>6.62	94.2<br>3.3	6.36<br>(8.15)<br>9.68	94.2<br>1.5<br>4.3
	**f/f0**<br>**rmsd**	10.4<br>0.380		1.6 (fix)<br>0.029	
90% monomer (6.33 S) +<br>10 % dimer (9.41 S)	**peaks**	4.38<br>6.21	92.8<br>3.4	6.28<br>9.32	89.6<br>10.2
	**f/f0**<br>**rmsd**	9.16<br>0.360		1.6 (fix)<br>0.034	
80% monomer (6.33 S) +<br>20 % dimer (9.41 S)	**peaks**	4.49<br>6.19	88.2<br>7.3	6.32<br>(7.99)<br>9.40	79.4<br>0.4<br>20.2
	**f/f0**<br>**rmsd**	8.29<br>0.318		1.6 (fix)<br>0.037	
