# Supplementary material for: Measuring macromolecular size distributions and interactions at high concentrations by sedimentation velocity
Source: Nat Commun. 2018 Oct 24;9:4415. doi: 10.1038/s41467-018-06902-x (PMC6200768; doi:10.1038/s41467-018-06902-x)
Supplement: Supplementary file 2 — Peer Review File [file 41467_2018_6902_MOESM2_ESM.pdf]

Reviewer #1 (Remarks to the Author):

The authors propose a new way of utilising standard AUC methods on commercially-available instruments to study macromolecules at high concentrations. At low concentrations, macromolecules can be studied by sedimentation velocity using size distribution plots c(s). At high concentrations, as in this study, the size distributions are still produced, but a new nonideality parameter is now described in order to make these important analyses possible.

This reviewer did have difficulty in following and understanding the manuscript, and establishing whether the major claims of the paper will be understood by the general AUC community. Reviewers are asked to "assess the clarity and context" of the manuscript, specifically whether the manuscript is clear and accessible, and whether the sections are appropriate. Even after re-reading this a few times, to try and make better sense of this, I am happy to agree that this work is suitable for Nature Communications, but it does need a major rewrite to make this accessible for the community, otherwise its significance will be lost. Examples (non-exhaustive) are listed:

Abstract – surely the essence of the new method needs to be explained here, rather than making vague statements about how useful the new method will be. The first seven lines of the abstract could be condensed to create the necessary space.

Introduction – comprised of three pages – some of which is not clear (eg: what does "This" refer to at the last sentence of paragraph 2?). What exactly is the end-product of the new procedure – does this involve a new cNI(s0) analysis (if so, please make this clearer), is this to do with Ks and Kd or is this B2? Why is the NISTmAb highly nonideal – it seems quite conventional (see top page 10)?

Methods – It is also not easy to follow the flow of argument in the first 2.5 pages of Methods. Probably the authors need to be more upfront about saying that cNI(s0) is the main output of the new method, and generate a clear flow of explanation.

Results – Fig 1 and Fig 2 seem incomplete. What residuals does the panel under the boundaries refer to? If Fig 2 follows from Fig 1, it seems easier for readability to merge both figures, as in Supp Fig 1. In Fig 2, why is the vertical axis c(s) and not cNI(s0) – this is confusing.

Can the authors determine kS for their very dilute mAb in Supp Fig 1 in order to compare this with the output in Fig 1 for 10 mg/ml mAb.

Text repetition – Eq 4 is cited twice on page 12 line 4.

Supp Fig 2. If the authors are using mg/ml units in most of their paper, it does not help to switch the units to M on the x-axis. See Supp Fig 5. Please explain how kS is determined from this figure.

Supp Fig 4 legend – do the authors mean Fig. 5 and not 3?

Reviewer #2 (Remarks to the Author):

Review of Nature Communications Manuscript ID: NCOMMS-18-16726: Measuring Macromolecular Size-Distributions and Interactions at High Concentrations by Sedimentation Velocity

This manuscript presents a new model for fitting sedimentation velocity (SV) data under non-ideal conditions for purposes of more accurately quantifying the size distribution and interactions of macromolecular species in solution at high concentrations. The algorithm provides a way to overcome the Johnston-Ogston effect, which is currently a problematic issue with SV analysis at high concentrations that can lead to non-trivial inaccuracies in SV measurements. The model also appears to provide an accurate and precise measure of the interaction parameter, ks. Overall, the authors make a convincing case of the accuracy and utility of their new fitting model, cNI(s0), and the authors' claims are well supported by the data presented in the paper.

I have only a few minor comments for the authors to consider:

1. The new model appears to provide artificially high values for the diffusion parameter, kd. The authors provided a brief hypothesis of the source of the ill-defined result, specifically, correlation between the polydispersity, frictional ratio parameter, and kd. Although the authors provide some limited discussion around potential mitigations to arrive at an accurate kd, a more thorough discussion would be beneficial. This additional discussion may include procedural techniques such as

a separate SE run to determine B2 from which kd can then be calculated (as the authors showed) or potentially future algorithm development.

2. For the NISTmAb experiments, the cNI(s0) model gave approximately 2.5% dimer for the dilute sample run in the 12 mm centerpiece and for the high concentration SV run in the 3 mm centerpieces. However, the model yielded a value of approximately 5% dimer using the 1.5 mm centerpiece. Although the authors suggest "reasonable consistency of results", in my experience, a difference between 2.5% and 5% dimer is significant. Additional (limited) discussion into the significance of this difference and the potential cause seems appropriate.

3. How were the simulated data in Figure 4 generated? The authors use simulated data to show the improvement of the new cNI(s0) model, but I suspect the data were generated using this new non-ideal model. If so, the manuscript should be clarified to state that the under-reporting of dimer obtained using the previous model is relative to the new model (not compared to an independent source of "truth"). The authors proceed to show the effect experimentally using BSA, but even so, they should consider providing more information about how the simulated data were generated.

4. There are a few minor typos throughout the document that should be corrected upon another careful review by the authors (e.g., equation 3 contains extra open parentheses before "1" in the diffusion expressions).

Overall, the paper is well written and concise, and it introduces a critical improvement to enable accurate analysis of protein size distributions and non-ideality parameters at high concentrations.

Reviewer #3 (Remarks to the Author):

Reviewer's Summary

The paper entitled "Measuring Macromolecular Size-Distributions and Interactions at High Concentrations by Sedimentation Velocity" written by Dr. Peter Schuck and this collaborators constitutes a very significant contribution to the area of macromolecular science that is greatly lacking in effective analytical tools capable of providing important details scientific information about macromolecular solution behavior at high concentrations. Overall the methodology outlined in this paper should open the door to many future papers dealing with this important topic and

further stimulate interest in the use of the analytical centrifugation for studying macromolecular solution behavior in a concentration range that is shrouded in a cloud missing knowledge. Nowhere more important is this need than in the area of molecular biology and biopharmaceutical development. Clearly, the sedimentation velocity methodology outlined in this paper bring us a quantum step closer to the real working range of living systems than that provided by biophysical studies conducted under idea solution conditions. In general I strongly support the publication of this paper. In doing so, however, this reviewer has some of the following recommendations, questions, and minor edits that I would like the authors to consider or address before the paper is finally approved and published, see below:

1. In the last sentence in the abstract consider the following change:

"It offers high resolution and sensitivity up to 50 mg/mL of protein, extending studies …." to "It offers high resolution and sensitivity on protein solutions up to 50 mg/mL, extending studies …."

2. On page 2, middle paragraph, 2nd sentence from the top, add "to" to the sentence as indicated below:

"In nonideal solutions the motion of each particle is modulated depending on the position of all others, due to the combined …."

3. On page 2, 2nd sentence from the top of the bottom paragraph, consider the following change:

"It also features exquisite sensitivity to trace populations, and allows gentle experimental conditions that causes negligible sample dilution and do not require any tag and lack surfaces." to "It also features exquisite sensitivity to trace populations, allows gentle experimental conditions that causes negligible sample dilution, requires no tag and results in minimal surface interactions."

4. On page 3, last sentence in the bottom paragraph, consider the following change:

"This extends high-resolution polydispersity analyses by 1 – 2 orders …." to "This method extends high-resolution polydispersity analyses via SV by 1 – 2 orders …."

5. On page 5 insert an “a” in the sentence that appears right after equation 4. It would read as follows:

“and is a function of the ….”

6. On page 5, I question the usage of “at the available scans” in the 2nd sentence from the bottom of the page, since experimental data corresponds to the available scans. Hence I would suggest making the following change:

“The match between the experimental data at the available scans and the calculated ….” to ““The match between the experimental and the calculated ….”

7. On page 6, at the end of the 1st sentence at the top of the page, consider adding the following text indicated below to help the reader understand that you are talking about the unique problems that arises from meniscus and buffer mis-matches between both the sample and reference sectors, which needs to be accounted for when the using interference detection that the reader may not find apparent by just saying “offset from sedimenting co-solutes”.

“…. and offset from sedimenting co-solutes (resulting from small meniscus and buffer composition mis-matches between sample and reference sectors).”

Note, in dealing with sedimenting co-solutes this reviewer is assuming that given the relatively low level and nature of buffer excipients used in this paper the accounting of viscosity and density gradients generated from the sedimentation of these co-solutes, excipients, during centrifugation is not required, is this correct?

8. In using this new method to characterize the NISTmAb at a concentration of 0.2 mg/mL and 10 mg/mL the authors report that the dimer level increased by a factor of 2 (from 2.5% to 5.3%), but make no comment on this reported difference. My question is, does this imply an increase in dimer formation due to concentration or does this imply something about the level of accuracy in the new method to determine trace aggregation?

9. In the Figure 5b insert, why is the same c(s) data provided for the dimer (via open circles) not provided for the trimer (via open upside down triangle)?

10. On the 2nd page of the discussion (page 15 of the paper), 2nd paragraph from the bottom, in the beginning of the last sentence consider the following change:

“More generally it unclear whether ….” to “More generally it is unclear whether ….”

11. In supplemental Figure 1 the 0.2 mg/mL sample of NISTmAb shows a starting OD reading for the sample of about 0.65 ODs at 280nm. However, the extinction coefficient of this molecule is stated to have value of 1.42 mg/mL/cm. Consequently, for a 1.2 cm centerpiece an actual OD280 reading would be 0.34, which is about a factor of 2 smaller than what is recorded by the analytical centrifuge UV detector! Although the AUC UV detector may not give identical OD values to that recorded by static tabletop spectrophotometry, a factor of 2 difference is alarming! Is there an error somewhere here? Please explain this observed difference to the reviewer.

12. It would be a good idea to help the reader by stating that the centerpiece used in Figures 1, 3 and supplemental Figures 1, 4 is a 3 mm centerpiece in the legend text. This would also be consistent with other Figures where centerpiece pathlength is given.

Reviewer 1

Reviewer #1: The authors propose a new way of utilising standard AUC methods on commercially-available instruments to study macromolecules at high concentrations. At low concentrations, macromolecules can be studied by sedimentation velocity using size distribution plots c(s). At high concentrations, as in this study, the size distributions are still produced, but a new nonideality parameter is now described in order to make these important analyses possible.

This reviewer did have difficulty in following and understanding the manuscript, and establishing whether the major claims of the paper will be understood by the general AUC community. Reviewers are asked to "assess the clarity and context" of the manuscript, specifically whether the manuscript is clear and accessible, and whether the sections are appropriate. Even after re-reading this a few times, to try and make better sense of this, I am happy to agree that this work is suitable for Nature Communications, but it does need a major rewrite to make this accessible for the community, otherwise its significance will be lost. Examples (non-exhaustive) are listed:

Our Response: We thank the author for recognizing the importance of this contribution, and for his/her suggestion to make it more accessible. Following the comments of this and the other two reviewers, we feel we have significantly increased the clarity of the presentation in the revised manuscript.

Reviewer #1: Abstract – surely the essence of the new method needs to be explained here, rather than making vague statements about how useful the new method will be. The first seven lines of the abstract could be condensed to create the necessary space.

Our Response: We believe that to understand the essence of the method a description of the background and scope of the problem is indispensable. However, in order to address the Reviewer's concern we have rephrased several sentences in the abstract, now emphasizing that the method can for the first time resolve macromolecular size-distributions at high concentrations, and that this is made possible by simultaneously accounting for mutual interactions. This is the essence of the method, and it concretely boils down to the fact that we can now analyze protein size distributions up to 50 mg/ml, up from previously 1-2 mg/ml in favorable cases.

Reviewer #1: Introduction – comprised of three pages – some of which is not clear (eg: what does "This" refer to at the last sentence of paragraph 2?).

Our Response: We have shortened the introduction, and eliminated the last sentence of paragraph 2.

Reviewer #1: What exactly is the end-product of the new procedure – does this involve a new cNI(s0) analysis (if so, please make this clearer), is this to do with Ks and Kd or is this B2?

Our Response: Yes, it does involve a new cNI(s0) analysis made possible by accounting for ks and kd. We have rephrased the last sentence in the second-to-last paragraph in the introduction to read "This makes possible a new size-distribution analysis method termed $c_{NI}(s_0)$ for polydisperse nonideal systems at high concentrations: It is a diffusion-deconvoluted sedimentation coefficient distribution that is approximately corrected for nonideality, and provides estimates for the average nonideality parameters for sedimentation and diffusion k_S and k_D."

Reviewer #1: Why is the NISTmAb highly nonideal – it seems quite conventional (see top page 10)?

Our Response: The reviewer must have misread our text "the highly nonideal solutions of NISTmAb reference material": The NISTmAb is of course a standard antibody, but, like any other protein, at high concentrations it will form a nonideal solution. 'Nonideal solution' is a standard expression in thermodynamics of macromolecular solutions to describe the fact that physical interactions occur between solutes, in contrast to 'ideal solutions'.

We have rephrased to make this more accessible: "We explore the performance of this new method with experimental data from the highly nonideal solutions of proteins at high concentrations, including suspensions of the NISTmAb reference material,…".

Reviewer #1: Methods – It is also not easy to follow the flow of argument in the first 2.5 pages of Methods. Probably the authors need to be more upfront about saying that cNI(s0) is the main output of the new method, and generate a clear flow of explanation.

Our Response: The first Methods section is entitled 'Nonideal Sedimentation Coefficient Distributions'. Following the Reviewer's suggestion, we inserted a new first sentence to describe the flow of what follows: "We describe in the following the mathematical structure of the new sedimentation coefficient distributions $c_{NI}(s_0)$, then summarize the description of sedimentation and diffusion in nonideal solutions, and finally show how $c_{NI}(s_0)$ and the nonideality parameters are computationally coupled and calculated."

Reviewer #1: Results – Fig 1 and Fig 2 seem incomplete. What residuals does the panel under the boundaries refer to? If Fig 2 follows from Fig 1, it seems easier for readability to merge both figures, as in Supp Fig 1. In Fig 2, why is the vertical axis c(s) and not cNI(s0) – this is confusing.

Our Response: We thank the reviewer for pointing out the lack of description for the residuals in Fig. 1. We have added: "The lower panel shows residuals of the $c_{NI}(s_0)$ fit." Also, we have fixed the ordinate of Fig. 2 to read "normalized c(s) or $c_{NI}(s_0)$". We decided not to merge Fig 1 and Fig 2 so that the details of the boundary fit in Fig. 1 can be better displayed without taking up too much page space for Fig 2. We envision Fig 1 to span 2 columns and Fig 2 to span a single column.

Reviewer #1: Can the authors determine kS for their very dilute mAb in Supp Fig 1 in order to compare this with the output in Fig 1 for 10 mg/ml mAb.

Our Response: No – the very dilute solution is close to ideal and does not exhibit significant nonideality, thus does not offer the possibility to determine the nonideality parameter of sedimentation k_S. However, the concentration dependence of the sedimentation coefficient at a range of concentrations can be used to determine k_S, and this was carried out and shown in Supp. Fig. 2. The manuscript reads: "To assess their validity, we carried out an independent measurement of k_S from the traditional regression of the weighted-average sedimentation coefficient as a function of loading concentration across a dilution series (**Supplementary Figure 2**)."

Reviewer #1: Text repetition – Eq 4 is cited twice on page 12 line 4.

Our Response: Thank you, we have eliminated the repetition.

Reviewer #1: Supp Fig 2. If the authors are using mg/ml units in most of their paper, it does not help to switch the units to M on the x-axis. See Supp Fig 5. Please explain how kS is determined from this figure.

Our Response: We thank the reviewer for pointing out this inconsistency in units – we have changed Supp Fig 2 to mg/ml units as in Supplementary Fig. 5 and the rest of the manuscript. We also have added a reference to Eq. 3 to both Supplementary Figure legends.

Reviewer #1: Supp Fig 4 legend – do the authors mean Fig. 5 and not 3?

Our Response: We have double checked the cross-references. Supp Fig 4 shows the boundary data of apoferritin, corresponding to the sedimentation coefficient distributions of Fig. 3, as stated.

Reviewer 2

Reviewer #2: This manuscript presents a new model for fitting sedimentation velocity (SV) data under non-ideal conditions for purposes of more accurately quantifying the size distribution and interactions of macromolecular species in solution at high concentrations. The algorithm provides a way to overcome the Johnston-Ogston effect, which is currently a problematic issue with SV analysis at high concentrations that can lead to non-trivial inaccuracies in SV measurements. The model also appears to provide an accurate and precise measure of the interaction parameter, ks. Overall, the authors make a convincing case of the accuracy and utility of their new fitting model, cNI(s0), and the authors' claims are well supported by the data presented in the paper.

Our Response: We thank the reviewer for positively recognizing the accuracy and utility of the new model.

Reviewer #2: I have only a few minor comments for the authors to consider:
1. The new model appears to provide artificially high values for the diffusion parameter, kd. The authors provided a brief hypothesis of the source of the ill-defined result, specifically, correlation between the polydispersity, frictional ratio parameter, and kd. Although the authors provide some limited discussion around potential mitigations to arrive at an accurate kd, a more thorough discussion would be beneficial. This additional discussion may include procedural techniques such as a separate SE run to determine B2 from which kd can then be calculated (as the authors showed) or potentially future algorithm development.

Our Response: We have expanded the discussion of this point. Briefly, in our opinion it is unclear (even doubtful) whether potential future extensions permitting study of even higher concentrations would help to resolve the correlation. However, as the reviewer indicates, the combination with other methods might be good strategy to circumvent the problem. The information on polydispersity provided by cNI(s0) should be helpful in this regard, to aid in the data interpretation of other techniques.

Reviewer #2: 2. For the NISTmAb experiments, the cNI(s0) model gave approximately 2.5% dimer for the dilute sample run in the 12 mm centerpiece and for the high concentration SV run in the 3 mm centerpieces. However, the model yielded a value of approximately 5% dimer using the 1.5 mm centerpiece. Although the authors suggest "reasonable consistency of results", in my experience, a

difference between 2.5% and 5% dimer is significant. Additional (limited) discussion into the significance of this difference and the potential cause seems appropriate.

Our Response: To address this question we have carried out a series replicate experiments and estimated precision of dimer fraction to be 0.8%. We have inserted this number in the results section. This confirms the Reviewer's suspicion that the difference is significant even for the $c_{NI}(s_0)$ analysis. We believe that this may exhibit a limitation of current 3D printed centerpieces: Since they are not as smooth and precise as the standard Epon centerpieces, low level convective disturbances may be the origin for the elevated dimer fraction, reminiscent of batches of Beckman Epon centerpieces reported by Gabrielson and colleagues in 2009. We have added two sentences in the third paragraph of the discussion, along with the new reference:

"3D printed centerpieces with shorter pathlength, as applied in the present work for comparison, have the potential to further increase the dynamic range. However, the dimer fraction of 5.3% measured for the NISTmAb using the 3D printed centerpieces is slightly elevated compared to the value of 2.7% obtained from samples in commercial Epon 3 mm centerpieces. This may possibly be due to imperfections in the 3D printed centerpieces causing convective disturbances, reminiscent of elevated dimer fractions observed in earlier, less precisely manufactured batches of commercial Epon centerpieces.[64] Optimized experimental strategies and concentration limits will be further explored elsewhere."

Reviewer #2: 3. How were the simulated data in Figure 4 generated? The authors use simulated data to show the improvement of the new cNI(s0) model, but I suspect the data were generated using this new non-ideal model. If so, the manuscript should be clarified to state that the under-reporting of dimer obtained using the previous model is relative to the new model (not compared to an independent source of "truth"). The authors proceed to show the effect experimentally using BSA, but even so, they should consider providing more information about how the simulated data were generated.

Our Response: We have clarified that the simulations were not based on the new model, but a much simpler model of non-ideal sedimentation for known discrete species, which has been around for many decades and has been validated against experimental data (e.g., ref 41): "Simulations were carried out using a conventional model for two discrete non-ideally sedimenting species with given parameters, and the resulting sedimentation patterns were analyzed with either standard $c(s)$ or the new $c_{NI}(s_0)$ method allowing for unknown distributions and interaction parameters in the inverse problem. "

Reviewer #2: 4. There are a few minor typos throughout the document that should be corrected upon another careful review by the authors (e.g., equation 3 contains extra open parentheses before "1" in the diffusion expressions).

Our Response: We have double checked the manuscript for typos, and we have removed the extra open parentheses in the second line of Eqs. 3.

Reviewer #2: Overall, the paper is well written and concise, and it introduces a critical improvement to enable accurate analysis of protein size distributions and non-ideality parameters at high concentrations.

Our Response: We thank the reviewer again for the positive assessment and careful reading.

Reviewer 3

Reviewer #3: The paper entitled “Measuring Macromolecular Size-Distributions and Interactions at High Concentrations by Sedimentation Velocity” written by Dr. Peter Schuck and this collaborators constitutes a very significant contribution to the area of macromolecular science that is greatly lacking in effective analytical tools capable of providing important details scientific information about macromolecular solution behavior at high concentrations. Overall the methodology outlined in this paper should open the door to many future papers dealing with this important topic and further stimulate interest in the use of the analytical centrifugation for studying macromolecular solution behavior in a concentration range that is shrouded in a cloud missing knowledge. Nowhere more important is this need than in the area of molecular biology and biopharmaceutical development. Clearly, the sedimentation velocity methodology outlined in this paper bring us a quantum step closer to the real working range of living systems than that provided by biophysical studies conducted under idea solution conditions. In general I strongly support the publication of this paper. In doing so, however, this reviewer has some of the following recommendations, questions, and minor edits that I would like the authors to consider or address before the paper is finally approved and published, see below:

Our Response: We are very happy the reviewer agrees with our view of the importance and potential impact of the new approach.

Reviewer #3: 1. In the last sentence in the abstract consider the following change:
“It offers high resolution and sensitivity up to 50 mg/mL of protein, extending studies ….” to “It offers high resolution and sensitivity on protein solutions up to 50 mg/mL, extending studies ….”

Our Response: We agree and have made this change.

Reviewer #3: 2. On page 2, middle paragraph, 2nd sentence from the top, add “to” to the sentence as indicated below:
“In nonideal solutions the motion of each particle is modulated depending on the position of all others, due to the combined ….”

Our Response: Thank you for pointing out this typo.

Reviewer #3: 3. On page 2, 2nd sentence from the top of the bottom paragraph, consider the following change:
“It also features exquisite sensitivity to trace populations, and allows gentle experimental conditions that causes negligible sample dilution and do not require any tag and lack surfaces.” to “It also features exquisite sensitivity to trace populations, allows gentle experimental conditions that causes negligible sample dilution, requires no tag and results in minimal surface interactions.”

Our Response: We agree this flows better and have made this change.

Reviewer #3: 4. On page 3, last sentence in the bottom paragraph, consider the following change:

“This extends high-resolution polydispersity analyses by 1 – 2 orders ….” to “This method extends high-resolution polydispersity analyses via SV by 1 – 2 orders ….”

Our Response: We have made this change.

Reviewer #3: 5. On page 5 insert an "a" in the sentence that appears right after equation 4. It would read as follows:
"and is a function of the …."

Our Response: Thank you, we have fixed this typo.

Reviewer #3: 6. On page 5, I question the usage of "at the available scans" in the 2nd sentence from the bottom of the page, since experimental data corresponds to the available scans. Hence I would suggest making the following change:

"The match between the experimental data at the available scans and the calculated …." to ""The match between the experimental and the calculated …."

Our Response: We agree - this was not a good statement. Since it adds little to the description of the method we have deleted the entire sentence.

Reviewer #3: 7. On page 6, at the end of the 1st sentence at the top of the page, consider adding the following text indicated below to help the reader understand that you are talking about the unique problems that arises from meniscus and buffer mis-matches between both the sample and reference sectors, which needs to be accounted for when the using interference detection that the reader may not find apparent by just saying "offset from sedimenting co-solutes".

"…. and offset from sedimenting co-solutes (resulting from small meniscus and buffer composition mis-matches between sample and reference sectors)."

Note, in dealing with sedimenting co-solutes this reviewer is assuming that given the relatively low level and nature of buffer excipients used in this paper the accounting of viscosity and density gradients generated from the sedimentation of these co-solutes, excipients, during centrifugation is not required, is this correct?

Our Response: The reviewer is correct. We have clarified the this statement and included an explanatory sentence: "Similarly, signal offsets from sedimenting co-solutes resulting from buffer composition mis-matches between sample and reference sectors can be included in the fit. (Modeling the impact on macromolecular sedimentation of dynamic density and viscosity gradients at high co-solute concentrations[new refs] is compatible with the current model but not yet implemented; this limits the application at present to sufficiently dilute buffer conditions.)"

Reviewer #3: 8. In using this new method to characterize the NISTmAb at a concentration of 0.2 mg/mL and 10 mg/mL the authors report that the dimer level increased by a factor of 2 (from 2.5% to 5.3%), but make no comment on this reported difference. My question is, does this imply an increase in dimer formation due to concentration or does this imply something about the level of accuracy in the new method to determine trace aggregation?

Our Response: We have measured a dimer fraction of 2.7 % at the same concentration of 10 mg/ml in commercial 3 mm centerpieces. This shows there is no concentration dependence.

The difference between the > 5% and 2.7% arises in the comparison of the 3D printed short-pathlength centerpieces and the commercial 3 mm Epon centerpieces (under otherwise identical conditions).

As detailed in the related comment by reviewer #2, we have carried out a series of replicate experiments to estimate the precision of the new method in determining dimer fractions. We found this to be approximately 1%. Therefore, the difference between the values obtained in the different centerpieces is real. We believe it is most likely be due to imperfections in the 3D printed centerpiece, possibly causing convective disturbances. This is reminiscent of elevated dimer fractions observed in older batches of commercial Epon centerpieces reported in 2009 by Gabrielson and colleagues. We have added two sentences at the end of the third paragraph of the discussion on this apparent current limitation of 3D printed centerpieces.

Reviewer #3: 9. In the Figure 5b insert, why is the same c(s) data provided for the dimer (via open circles) not provided for the trimer (via open upside down triangle)?

Our Response: Given that the fit of standard c(s) is extremely poor, in our judgement the interpretation of the much lower percentage trimer fractions would not be reasonable, since they describe features comprising smaller signals than the misfit of the data. We have added the caveat of interpreting c(s) traces from analyses where the model does not fit the data: "– to the extent that the resulting apparent distribution can be considered meaningful –".

Reviewer #3: 10. On the 2nd page of the discussion (page 15 of the paper), 2nd paragraph from the bottom, in the beginning of the last sentence consider the following change:

"More generally it unclear whether …." to "More generally it is unclear whether …."

Our Response: Thank you for pointing out this typo. The entire sentence was replaced with a more extensive discussion of the possibility for k_D determination as prompted by Reviewer 2.

Reviewer #3: 11. In supplemental Figure 1 the 0.2 mg/mL sample of NISTmAb shows a starting OD reading for the sample of about 0.65 ODs at 280nm. However, the extinction coefficient of this molecule is stated to have value of 1.42 mg/mL/cm. Consequently, for a 1.2 cm centerpiece an actual OD280 reading would be 0.34, which is about a factor of 2 smaller than what is recorded by the analytical centrifuge UV detector! Although the AUC UV detector may not give identical OD values to that recorded by static tabletop spectrophotometry, a factor of 2 difference is alarming! Is there an error somewhere here? Please explain this observed difference to the reviewer.

Our Response: We are grateful for the Reviewer to discover this discrepancy, and apologize for the confusion: The data shown wasn't absorbance but indeed interference data. We have fixed the ordinate label and legend. At 0.2 mg/ml and ~3.3 fringes/mg/ml the starting signal of ~0.67 fringes is consistent with the expectation.

Reviewer #3: 12. It would be a good idea to help the reader by stating that the centerpiece used in Figures 1, 3 and supplemental Figures 1, 4 is a 3 mm centerpiece in the legend text. This would also be consistent with other Figures where centerpiece pathlength is given.

Our Response: We have added information on which centerpiece was used in Figure 1, Supplementary Figures 1, 4, and 7, to make sure that all figures showing raw data now have this information.

Reviewer #1 (Remarks to the Author):

I have read all the comments from the authors and I am happy to approve the corrected manuscript.

Reviewer #2 (Remarks to the Author):

I thank the authors for carefully addressing my comments and those of the other referees. I have no further comments on the manuscript.

Reviewer #3 (Remarks to the Author):

On reviewing the revised manuscript this reviewer is more than satisfied with how the authors have addressed my initial comments and suggested edits. As I stated in my initial review I find this paper making a significant contribution to the science of macromolecular solution behavior that is important to a wide range of scientific topics, most important of which is the behavior of biological macromolecules such as proteins and their self-assembly into nano- or supra-molecular structures that are involved in creating and controlling a living system and employed as biopharmaceuticals. I highly endorse the approval of this paper. It may not necessarily be a ground breaking paper (only time will tell) to this complex subject, but it certainly opens the door experimentally and theoretically to an approach that can provide useful information and an approach that can be further developed and critically assessed by others via future scientific work.

Two additional minor points and a possible correction that I would like to further suggest to the authors are the following:

1. Once "sedimentation velocity analytical ultracentrifugation" is defined as "SV", in the 2nd sentence of the bottom paragraph on the 2nd page of the introduction, why repeat the process of defining SV several times in the manuscript (see under the heading of "Analytical Centrifugation" 7th sentence down & under "Discussion" 2nd sentence down). I would take that 2nd sentence on the bottom paragraph of the 2nd page in the introduction and change it to read "solution, analytical ultracentrifugation (AUC) using sedimentation velocity (SV) stands out … ". By doing this the authors

could also remove the need to define "AUC" under the heading of "Analytical Centrifugation" in the 1st sentence of this section so this 1st sentence would just read "AUC experiments were carried …".

2. In the introduction I would place the 1st sentence in the 2nd paragraph into the last sentence of the 1st paragraph so that this last sentence in the 1st paragraph would read as "The balance of these forces can control an amazingly wide range of behaviors, which is well illustrated in our current understanding of cell biology."

3. In the Supplemental Material in Figure legend 1, I believe "deviation (rmsd) of 0.0045 OD" should be "deviation (rmsd) of 0.0045 fringes" given the stated use of the Rayleigh Interferometer.

Reviewer #1 (Remarks to the Author):

I have read all the comments from the authors and I am happy to approve the corrected manuscript.

Our Response: We thank the reviewer for his approval.

Reviewer #2 (Remarks to the Author):

I thank the authors for carefully addressing my comments and those of the other referees. I have no further comments on the manuscript.

Our Response: We thank the reviewer again for his constructive comments and are happy to see our revision finds the reviewer's agreement.

Reviewer #3 (Remarks to the Author):

On reviewing the revised manuscript this reviewer is more than satisfied with how the authors have addressed my initial comments and suggested edits. As I stated in my initial review I find this paper making a significant contribution to the science of macromolecular solution behavior that is important to a wide range of scientific topics, most important of which is the behavior of biological macromolecules such as proteins and their self-assembly into nano- or supra-molecular structures that are involved in creating and controlling a living system and employed as biopharmaceuticals. I highly endorse the approval of this paper. It may not necessarily be a ground breaking paper (only time will tell) to this complex subject, but it certainly opens the door experimentally and theoretically to an approach that can provide useful information and an approach that can be further developed and critically assessed by others via future scientific work.

Our Response: We are very pleased by the encouraging comments.

Two additional minor points and a possible correction that I would like to further suggest to the authors are the following:

1. Once "sedimentation velocity analytical ultracentrifugation" is defined as "SV", in the 2nd sentence of the bottom paragraph on the 2nd page of the introduction, why repeat the process of defining SV several times in the manuscript (see under the heading of "Analytical Centrifugation" 7th sentence down & under "Discussion" 2nd sentence down). I would take that 2nd sentence on the bottom paragraph of the 2nd page in the introduction and change it to read "solution, analytical ultracentrifugation (AUC) using sedimentation velocity (SV) stands out … ". By doing this the authors could also remove the need to define "AUC" under the heading of "Analytical Centrifugation" in the 1st sentence of this section so this 1st sentence would just read "AUC experiments were carried …".

Our Response: We have adopted this change and eliminated the duplications of the definitions for AUC and SV.

2. In the introduction I would place the 1st sentence in the 2nd paragraph into the last sentence of the 1st paragraph so that this last sentence in the 1st paragraph would read as "The balance of these forces can control an amazingly wide range of behaviors, which is well illustrated in our current understanding

of cell biology.”

Our Response: We have rearranged the sentences as suggested.

3. In the Supplemental Material in Figure legend 1, I believe “deviation (rmsd) of 0.0045 OD” should be “deviation (rmsd) of 0.0045 fringes” given the stated use of the Rayleigh Interferometer.

Our Response: We have corrected this and thank the reviewer for improving the manuscript.
